# Structural Conservation of the A_1_ Binding Site in Photosystem I across Cyanobacteria and Green Algae

**DOI:** 10.1021/acsomega.5c12267

**Published:** 2026-03-25

**Authors:** Gary Hastings, Hiroki Makita, Neva Agarwala, Michael R. Nelson, Julia S. Kirpich, Komalpreet Singh, Sreeja Parameswaran, Fedaa Ali, Barry D. Bruce, Haijun Liu, Lujun Luo, Wu Xu, Kevin Redding, Claudia Schade, Sarah M. Mäusle, Dennis J. Nürnberg

**Affiliations:** † Department of Physics and Astronomy, 1373 Georgia State University, Atlanta, Georgia 30303, United States; ‡ Molecular Biophysics and Integrated Bioimaging Division, Lawrence Berkeley National Laboratory. Berkeley, California 94720-8099, United States; § Department of Biochemistry & Cellular and Molecular Biology, 4292 University of Tennessee, Knoxville. Knoxville, Tennessee 37996-0840, United States; ∥ Program in Genome Science and Technology, Bredeson Center, University of Tennessee, Knoxville, Knoxville, Tennessee 37996-0840, United States; ⊥ Department of Biology, 7547 Saint Louis University, St. Louis, Missouri 63103-2097, United States; # Department of Chemistry, 4365 University of Louisiana at Lafayette, Lafayette, Louisiana 70503, United States; ∇ School of Molecular Sciences, Arizona State University, Tempe, Arizona 85287, United States; ○ Department of Physics, 9166 Freie Universität Berlin, Berlin 14195, Germany; ◆ Dahlem Centre of Plant Sciences, Freie Universität, Berlin 14195, Germany

## Abstract

Time-resolved step-scan Fourier transform infrared (FTIR) difference spectroscopy was used to obtain (A_1_^–^ – A_1_) FTIR difference spectra from photosystem I (PSI) samples isolated from eight phylogenetically diverse cyanobacterial strains and one green alga, totaling 13 PSI preparations. These included samples from cells grown under far-red light and PSI in monomeric, dimeric, trimeric, and tetrameric states. Spectral profiles were shown to be independent of oligomeric state. Remarkably, all (A_1_^–^ – A_1_) FTIR difference spectra exhibited high similarity, underscoring the robustness of the technique and indicating minimal experimental variability. This congruence reveals a highly conserved environment for the phylloquinone cofactor at the A_1_ binding site across diverse taxa. Conserved bands associated with the A_0_ pigment further suggest structural continuity from A_0_ to A_1_. To leverage this consistency, we constructed a composite (A_1_^–^ – A_1_) FTIR difference spectrum by averaging all 13 spectra. This composite spectrum provides enhanced resolution, enabling unambiguous identification of previously unresolved bands. The fact that a highly resolved composite spectrum can be obtained by averaging demonstrates the similarity in the spectra from the different types of samples. Band assignments were refined using prior studies, yielding an improved spectral framework for future investigations of PSI electron transfer cofactors.

## Graphical abstract

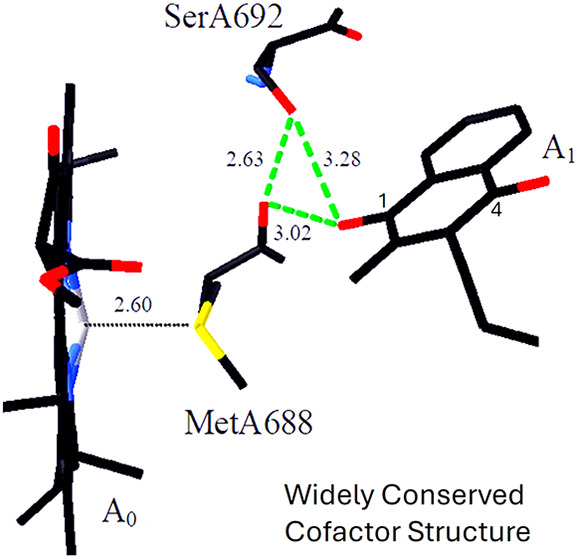

## Introduction

In photosynthesis solar energy is harvested and used to synthesize chemical products that form the foundation for most of the food and fuels consumed by humanity.^1^ In oxygen evolving photosynthetic organisms two photosystems – photosystem I (PSI)[Fn fn1] and photosystem II (PSII) – operate in tandem to absorb and transform light energy.^2^ Here we focus on PSI. Functionally, PSI acts as a light-driven enzyme that generates reducing power, which fuels the assimilation of carbon dioxide into complex organic molecules. In this way PSI plays a pivotal role in sustaining the biosphere and driving the global carbon cycle.

Solar energy conversion in PSI is initiated within a specialized pigment–protein complex known as the reaction center (RC). Upon light absorption, electrons are transferred sequentially via a series of pigment acceptors across the thylakoid membrane, a process central to biological energy transduction. The electron transfer (ET) cofactors (pigments) are coordinated by two membrane-spanning protein subunits called PsaA and PsaB.^3^ The spatial arrangement of these cofactors within PSI is illustrated in Figure 1A. The A and B subscripts in Figure 1A denote the ET branches rather than the specific protein subunit to which each pigment is bound.

**Figure 1:**
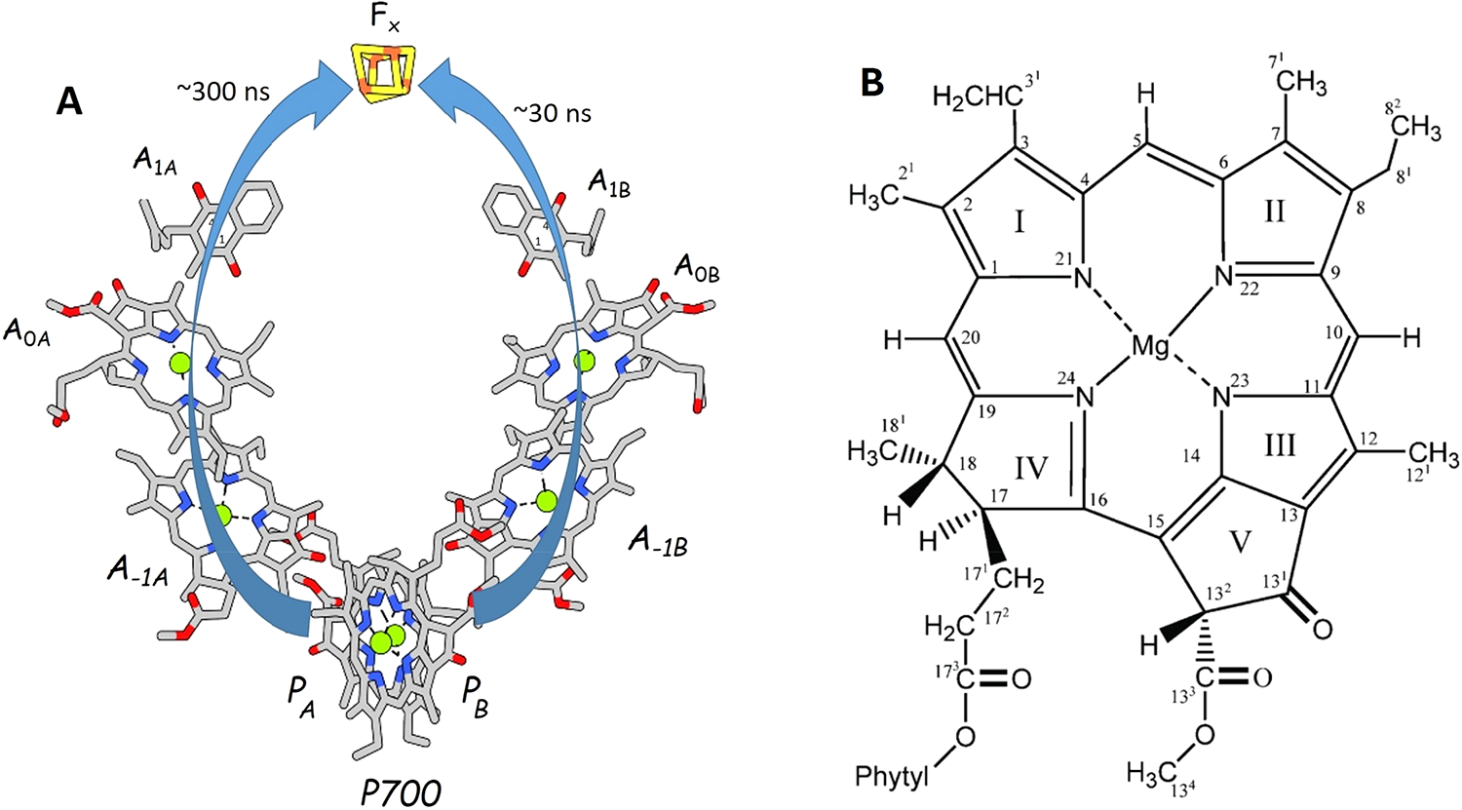
(A) Arrangement of ET cofactors in PSI from *Thermosynechococcus vestitus* BP-1 (*TV*). Cofactor hydrocarbon tails are truncated. ET pathways down the A and B branch are indicated. Time constants for A_1_^–^ to F_x_ ET at RT are also indicated. (B) Structure and IUPAC numbering scheme for Chl *a*.

At the heart of PSI is a pigment species called P700 that connects light harvesting to ET. P700 is often considered to be a heterodimeric chlorophyll *a*/chlorophyll *a*′ (Chl *a*/Chl *a*′) special pair.^4^ The P_B_/P_A_ pigment (Figure 1A) is a Chl *a*/Chl *a*′ species, respectively.^5^ Chl *a*′ is a 13^2^ epimer of Chl *a*.^6^ See Figure 1B for structure and numbering of Chl *a*.

Other work has called into question exactly which Chl’s may be the true primary electron donor, however, and it has been suggested that the P_B_/P_A_ pigments may be the secondary electron donor.^7^ Another idea is that P700 is a tetrameric Chl *a* species (consisting of P_A_, P_B_ A_–1A_ and A_–1B_), or at least four pigments are required to describe its spectroscopic properties.^8^ To complicate things further, it has also been proposed that one or more of the A_–1_ pigments is a Chl *f* molecule in PSI strains obtained from cells grown under far-red light (FRL).^9,10^ Chl *f* has a formyl group at the 2-position, respectively.^11^

In Figure 1A the A_0_ electron acceptor is a monomeric Chl *a* molecule.^5^ A_1_ is a phylloquinone (PhQ) molecule (also known as vitamin K_1_).^3,12,13^ PhQ is a 2-methyl, 3-phytyl, 1,4-naphthaquinone (Figure 5). Often the quinone in the A_1_ binding site is referred to as A_1_. Here the quinone in the binding site will be referred to by name, while the term A_1_ will refer to the actual binding site, thus PhQ occupies the A_1_ binding site in native PSI.

In isolated PSI, following light excitation, the P700^+^A_1_^–^ radical pair state is formed within 50 ps.^14,15^ Subsequently, an electron is transferred from A_1_^–^ to F_X_ and then on to F_A_ and F_B_ (this latter ET to F_A_ and F_B_ is not shown in Figure 1A). F_X_, F_A_ and F_B_ are iron sulfur clusters.^3^ At room temperature (RT, ∼ 295 K) ET from A_1_^–^ to F_X_ occurs in ∼30 or 300 ns (Figure 1A).^16,17^ The 30/300 ns time constants represent ET down the B/A branches, representing (A_1B_^–^ to F_X_)/(A_1A_^–^ to F_X_) ET, respectively.^18–21^ B-branch forward ET is an order of magnitude more rapid than on the A-branch. The structural basis for this difference is not well understood.^22–25^

At RT, ET from F_X_^–^ to F_A_ and then on to F_B_ occurs in ∼100 ns.^26^ In the absence of added electron acceptors, the P700^+^F_A/B_^–^ state recombines in ∼80 ms.^3^ P700^+^F_A/B_ ® recombines via repopulation of the P700^+^A_1_^–^ state.^27^ The F_A/B_ terminology is used to indicate that we do not distinguish between the F_A_ and F_B_ states.

Following light excitation of PSI at 77 K, in a fraction of the RCs, ET beyond A_1_ is inhibited and the P700^+^A_1_^–^ state undergoes a recombination reaction characterized by a time constant of ∼350 μs at 77 K in cyanobacterial PSI.^16,20^ In PSI at 77 K ET occurs almost exclusively along the A-branch.^28,29^ Therefore, repetitive-flash microsecond time-resolved (TR) experiments at 77 K in PSI preferentially probes the P700^+^A_1A_^–^ radical pair state. Microsecond time-resolved IR experiments on large photosynthetic proteins (such as PSI), are relatively easy to implement with high sensitivity using the step-scan approach,^30–32^ and we have produced microsecond time-resolved step-scan (TRSS) (P700^+^A_1A_^–^ – P700A_1A_) FTIR difference spectra (DS).

In PSI at 77 K, the P700^+^F_X_^–^ state is formed in a portion of the PSI RCs, and this state recombines over the course of several milliseconds at 77 K.^20^ The lifetime of the P700^+^F_X_^–^ state at 77 K is sufficiently long so that a portion of this radical pair state can be accumulated under continuous illumination, allowing the production of photoaccumulated (P700^+^F_X_^–^ – P700F_x_) FTIR DS. F_X_ does not contribute significantly in the spectral region of interest (1800–1400 cm^–1^) and this latter DS is often considered to be a (P700^+^ – P700) FTIR DS.

By subtracting the TR (P700^+^A_1A_^–^® – the P700A_1A_) FTIR DS from the photoaccumulated (P700^+^ – P700) FTIR DS we have shown that contributions from P700 and P700^+^ are effectively removed and the resulting spectrum is referred to as an (A_1_^–^ – A_1_) FTIR DS.^31,33^

In the past we have used TRSS FTIR DS to produce (A_1_^–^ – A_1_) FTIR DS for unlabeled and isotope labeled native PSI samples at 77 K.^34^ We have also produced (A_1_^–^ – A_1_) FTIR DS for PS I particles that have a modified PhQ species occupying the A_1_ binding site,^35^ or a completely different quinone species in the binding site.^36^ These quinone replacement studies were undertaken using PS I samples from a *menB* ^–^ mutant of *Synechocystis* sp. PCC 6803 (*S6803*).^37^

Based on the (A_1_^–^ – A_1_) FTIR DS we have obtained for PSI particles from *S6803* under a variety of conditions, we have proposed assignments for many of the bands in the FTIR DS.^33^ The question to be addressed in this manuscript is whether the FTIR DS are the same or similar in PSI from different strains. Furthermore, even in the FTIR DS obtained for PSI from *S6803*, there are still lingering doubts and ambiguities about whether certain bands in the spectra are sufficiently above the noise level to be reliable. This is especially the case for FTIR DS in the amide I spectral region (1680–1630 cm^–1^). With the large number of new spectra presented here we can accurately assess experimental variability.

Here we present (A_1_^–^ – A_1_) FTIR DS at 77K obtained from a total of 13 different PSI samples originating from eight cyanobacterial strains and one green alga. By averaging the 13 DS it is shown to be possible to generate a highly resolved DS, allowing the observation and analysis of even weak bands in the DS. The fact that the 13 DS can be averaged to produce a highly resolved spectrum demonstrates considerable similarity in the DS for the different samples.

## Materials and Methods

Trimeric and monomeric PSI particles from *S6803* were prepared in the Liu lab using a strain in which CP47 is C-terminally His_6_-tagged (strain HT-3).^38^ Briefly, a bead-beater was used to break the cells on ice. The His_6_-tagged PSII was removed using a Ni-NTA affinity column^39^ and the flowthrough was loaded onto a sucrose gradient,^40^ which was ultracentrifuged at 205,000*g* for 12 h. The lower and upper green bands were for trimeric and monomeric PSI, respectively.^40^ The green bands were collected and resuspended in Tris-buffer containing 50 mM Tris, 0.04% (w/v) n-dodecyl−β–D-maltoside (β–DM), at pH 8, and stored at −80 C until use. Absence of PSII in the isolated PSI samples was verified using 77 K fluorescence spectrophotometry. Trimeric PSI particles from *S6803* were also prepared in the Xu^41^ and Hastings^14^ laboratories as described previously.

Trimeric PSI particles isolated from *menB* ^–^ mutant cells from *S6803* were prepared as described,^42^ and PhQ was added also as described.^43^

Whole cells from *Synechococcus* sp. PCC 7002 (*S7002*) were originally provided by Donald Bryant’s lab, and trimeric PSI particles were prepared in the Hastings lab as described.^44^

Cells from *Synechococcus elongatus* PCC 7942 (*S7942*) were grown in the Hastings lab, and PSI was prepared following the protocol described for *S6803*.^45^

Trimeric PSI particles from *Thermosynechococcus vestitus* BP-1 (*TV*) were prepared in the laboratories of Nürnberg, Kern and Bruce using procedures similar to that which have been described.^17^

PSI particles from *Fischerella thermalis* (*FT7521*) cells grown under white light (WL) and far-red light (FRL) were provided by Donald Bryant and prepared as described.^10^

PSI particles from *Chroococcidiopsis thermalis* PCC 7203 (*CT7203*) cells grown under WL and FRL were prepared in the Nürnberg lab using a chromatography approach. Cells were grown in BG11 ^46^ at 28 °C in a 25 L photobioreactor (xCubio; bbi-biotech GmbH) equipped with fluorescent lamps (Osram Lumilux DE LUXE 30*W*/930) for WL or 750 nm LEDs (Shenzhen GOULY LED) for FRL. Cells were harvested by centrifugation at 3000*g* for 5 min, frozen in liquid nitrogen and stored at −80 °C until use. For the isolation of thylakoid membranes, the cells were thawed on ice and resuspended in TBB (50 mM MES-NaOH, pH 6.0, 10% (v/v) glycerol, 1.2 M betaine, 5 mM MgCl_2_ and 5 mM CaCl_2_). After incubation for 1 h on ice, 1 mM benzamidine, 1 mM ε-amino-n-caproic acid and ∼6 mg of DNase I were added and further incubated for 10 min. Cells were lysed by using a continuous flow cell disruptor (Constant Systems) and three or five passages at 1.7 bar for FRL or WL cells, respectively. After centrifugation at 5000*g* and 4 °C for 10 min, the supernatant was centrifuged for 45 min at 180,000 x *g* and 4 °C. The pelleted thylakoid membranes were resuspended in TSB (50 mM MES-NaOH, pH 6.0, 10% (v/v) glycerol, 1.2 M betaine, 5 mM MgCl_2_ and 20 mM CaCl_2_) and solubilized at a chlorophyll concentration of 1 mg mL^–1^ with 1% (w/v) β–DM for 10 min at 4 °C. After an additional centrifugation at 180,000*g* for 30 min at 4 °C, the supernatant was loaded onto a Toyopearl DEAE-650S column equilibrated with buffer A (50 mM MES-NaOH, pH 6.0, 10% (v/v) glycerol, 1.2 M betaine and 20 mM CaCl_2_). After washing with one column volume of buffer A containing 7.5 mM 15 mM and 22.5 mM MgSO_4_, proteins were eluted with a linear gradient from 22.5 mM to 52.5 mM MgSO_4_ over 6.6 column volumes. The PSI fraction was collected, concentrated using an Amicon Ultra 100 kDa centrifugal filter and applied to a second Toyopearl DEAE-650S column as specified above. For the resulting PSI fraction the buffer was exchanged to buffer A using the aforementioned centrifugal units and the PSI frozen in liquid nitrogen and stored at −80 °C until use.

PSI from WL grown *CT7203* cells were initially tetrameric^47^ but fell apart into dimers during the isolation procedure. PSI from FRL-grown cells remained trimeric as previously observed.^47^

Tetrameric PSI particles from *Chroococcidiopsis* sp. TS-821 (*CTS821*) were prepared as described previously.^48^

Green algal PSI from *Chlamydomonas reinhardtii* (*CR*) were prepared in the Redding lab as described.^49^

Tetrameric PSI samples from the cyanobacterium *Leptolyngbya ohadii* (*LO*) were prepared as described previously.^50^

For FTIR DS experiments PSI samples were washed in buffer and ultracentrifuged at 408,000*g* for 3 h to produce a soft pellet with reduced water content. 0.1 μL each of sodium ascorbate (20 mM) and phenazine methosulfate (10 μM) were added to the soft pellet as exogenous electron donor to P700^+^. The pellet was squeezed between two 1-in. calcium fluoride windows with the thickness adjusted so that the absorption at ∼1656 cm^–1^ was less than 1.0 (at 295 K). Samples were loaded into a liquid nitrogen cryostat (Oxford Instruments, Santa Barbara, CA) and cooled to 77 K.

TR and photoaccumulated FTIR DS experiments were conducted using a Vertex80 FTIR spectrometer from Bruker Optics, (Bruker Optics, Billerica, MA). For photoaccumulation experiments, actinic light from a 15 mW helium–neon laser was used. Photoaccumulated FTIR DS are referred to as (P700^+^ – P700) FTIR DS, and these were obtained as described previously.^10^

For TRSS FTIR DS experiments, a ∼5 ns pulse at 532 nm from a Minilite Nd:YAG laser (Continuum, Santa Clara CA), operated at 10 Hz, was used as actinic light source. TRSS FTIR DS were collected for PSI samples at 77 K, as described previously.^51^ FTIR DS were collected in the 2005–1100 cm^–1^ region at 4 cm^–1^ resolution. TR data were collected in 6 μs increments, over a 3.5 ms time-range. In most cases ∼ 50 TRSS FTIR DS experiments are undertaken and the results averaged. From this averaged file usually the third to twelfth time points after the laser flash (18–72 μs) were averaged and this spectrum is considered to be a (P700^+^A_1_ ® – P700A_1_) FTIR DS. In this average DS a short-lived (∼15 μs) sample heating artifact (Figure S2) is effectively subtracted. (A_1_^–^ – A_1_) FTIR DS were produced by subtracting photoaccumulated (P700^+^ – P700) FTIR DS from the TR (P700^+^A_1_^–^ – P700A_1_) FTIR DS.

## Results

Figure 2 shows TRSS (P700^+^A_1_^–^ – P700A_1_) FTIR DS in the 1760–1400 cm^–1^ region, obtained using PSI samples from *TV* at 77 K (*black*). The DS shown is the average of three spectra obtained from samples prepared in different laboratories (Nürnberg, Bruce and Kern laboratories). The error bars (plus or minus) represent the standard error in the three measurements. The spectrum shown is the average of nine spectra collected in 6 μs increments after a laser flash. Usually, the first two or three DS after the flash are ignored as these contain contributions from a sample heating artifact (Figure S2). TRSS FTIR DS can roughly be considered as a FTIR DS collected at, on average, 45 μs after laser flash excitation. Photoaccumulated (P700^+^ – P700) FTIR DS at 77 K for PSI from *TV* are also shown (*red*). The DS are again the average of the three samples from the different laboratories, and the error bars are also shown.

**Figure 2:**
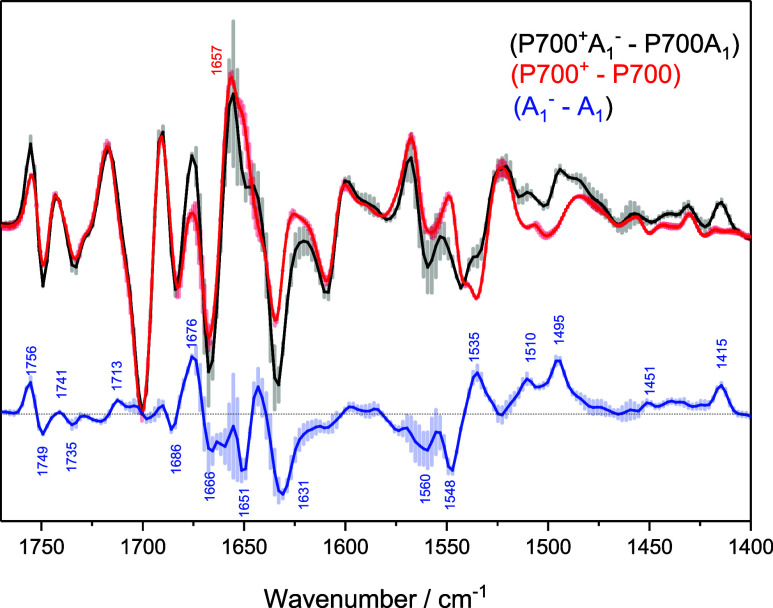
TRSS (P700^+^A_1_^–^ – P700A_1_) FTIR DS (*black)* obtained using PS I from *TV* at 77 K. The DS is the average of three spectra obtained from samples from different groups, and the error bars represent the standard deviation in the three measurements. The photoaccumulated (P700^+^ – P700) FTIR DS (*red*) obtained using PS I from *TV* at 77 K is also shown. Subtracting the TRSS FTIR DS from the photoaccumulated FTIR DS results in a (A_1_^–^ – A_1_) FTIR DS (*blue*). The propagated error bars are shown in the latter.

By subtracting the photoaccumulated FTIR DS from the TR FTIR DS, a relatively pure (A_1_^–^ – A_1_) FTIR DS can be obtained. The result of such a subtraction is also shown in Figure 2 (*blue*). The propagated error bars are also presented. One implicit assumption is that the spectrum of P700/P700^+^ is the same in both TR and photoaccumulation experiments. A second assumption is that F_X_/F_X_^–^ does not contribute to the FTIR DS in the spectral region considered,^33,52^ although weak spectral features that could potentially be related to F_X_ reduction were proposed recently.^53^

In a recent publication^17^ we presented (P700^+^ – P700) FTIR DS at both 77 and 295 K for PSI from *TV*. We also presented (P700^+^A_1_^–^ – P700A_1_) FTIR DS for this strain. To the best of our knowledge no other TR or photoaccumulated FTIR DS have been presented for *TV* PSI samples, and (A_1_^–^ – A_1_) FTIR DS for *TV* PSI are shown here for the first time. Within the error bars in Figure 2, the *TV* samples from the three different laboratories are identical.

Most of the bands in the (A_1_^–^ – A_1_) FTIR DS in Figure 2 are well above the noise level indicated by the error bars, except in the ∼1650–1670 cm^–1^ region. Despite the increased noise in this region, we tentatively suggest negative bands at 1651 cm^–1^ and 1666 cm^–1^, and a positive feature may also be present near 1657 cm^–1^.

Figure S1 compares (A_1_^–^ – A_1_) FTIR DS obtained for PSI from *TV* and *S6803*. The *S6803* DS is the average of three DS obtained from different groups (Bruce, Xu and Liu laboratories). The DS for *S6803* and *TV* share many similarities. A detailed (A_1_^–^ – A_1_) FTIR DS for PSI from *S6803* was presented in a review article^33^ and is similar to that shown in Figure S1 except the intensity ratio of the negative bands at 1561 and 1548 cm^–1^. These bands show some variability due to features associated with laser-induced sample heating that predominate in this region (Figure S2).^32,55–57^

Figure S1 also shows an (A_1_^–^ – A_1_) FTIR DS obtained for PSI from *S7002*. Figure S1 demonstrates similar (A_1_^–^ – A_1_) FTIR DS for PSI from *S7002, S6803* and *TV*. This similarity in DS extends to other strains, and Figure 3 shows (A_1_^–^ – A_1_) FTIR DS from PSI from 13 different PSI samples. Twelve from cyanobacteria and one from a green alga.

**Figure 3:**
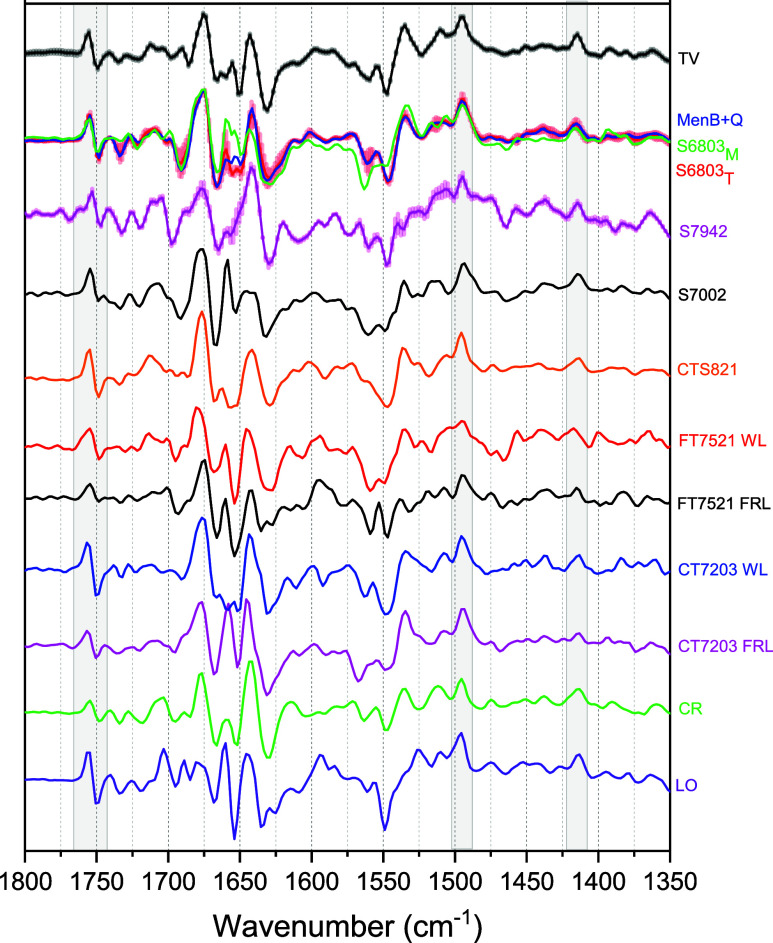
Comparison of (A_1_^–^ – A_1_) FTIR DS obtained using 13 different types of PS I samples. Twelve from cyanobacteria and one from green algae (*CR*). Samples included monomeric, dimeric, trimeric and tetrameric PSI. Two strains of PSI were from cells grown under FRL and WL (*CT7203* and *FT7521*).

Figure 3 compares FTIR DS obtained for monomeric and trimeric PSI samples from *S6803*, referred to S6803_M_ and S6803_T_, respectively. These FTIR DS are also compared to (trimeric) PSI samples obtained from *menB* ^–^ mutants of *S6803* where PhQ has been reincorporated back into the A_1_ binding site (denoted as MenB+Q). In *menB* ^–^ mutant PSI, a plastoquinone molecule normally occupies the A_1_ binding site. As has been demonstrated previously, DS for *menB* ^–^ PSI from *S6803* with PhQ incorporated are similar to DS obtained for trimeric PSI from *S6803*.^33^ There are some minor differences in the FTIR DS obtained for monomeric and trimeric PSI samples, particularly in the neutral quinone region, from ∼1630–1650 cm^–1^. There are also differences near 1560 and 1533 cm^–1^. As suggested above, and in Figure S2, these differences are likely related to differences in a laser flash induced sample heating artifact.^55–58^ The heating artifact has a time constant of ∼ 15 μs at 77 K, and displays intense bands at ∼1561(−) and 1664­(+) cm^–1^ (Figure S2).^55,56^

An (A_1_^–^ – A_1_) FTIR DS from PSI from *S7942* is shown in Figure 3 for the first time. This DS shares similarities with that obtained for PSI from *S6803* and *TV*. The FTIR DS for PSI from *S7002* in Figure 3 is similar to that presented previously,^31,34^ but here it is shown with higher signal-to-noise ratio, as indicated by the error bars in the expanded view of the DS in Figure S1.

Figure 3 shows further (A_1_^–^ – A_1_) FTIR DS obtained for PSI samples from *FT7521* obtained from cells grown under WL and FRL. These WL- and FRL-PSI samples from *FT7521* cells were the subject of a previous (P700^+^ – P700) FTIR DS study.^10^ TR (A_1_^–^ – A_1_) FTIR DS at 77 K have never been presented before for these FRL- and WL-PSI samples. The WL- and FRL-PSI samples from *FT7521* are both trimeric.^59^

Figure 3 also shows (A_1_^–^ – A_1_) FTIR DS obtained for PSI samples from *CT7203* obtained from cells grown under WL and FRL. Unlike that for PSI from *FT7521*, the FRL-PSI samples from *CT7203* are trimeric, while the WL-PSI samples are dimeric,^47^ probably arising from a tetrameric PSI in whole cells that is degraded to a dimer upon PSI purification/isolation.

Figure 3 also shows (A_1_^–^ – A_1_) FTIR DS obtained for PSI samples from *Chroococcidiopsis* sp. TS-821 (*CTS821*). These PSI samples from *CTS821* are well-known to be tetrameric.^48^ The (A_1_^–^ – A_1_) FTIR DS is also shown for PSI samples from *Leptolyngbya ohadii* (*LO*). *LO* PSI samples are also known to be tetrameric.^50^

Finally, Figure 3 shows (A_1_^–^ – A_1_) FTIR DS obtained for green-algal PSI samples from *Chlamydomonas reinhardtii* (*CR*). This is the first example of (A_1_^–^ – A_1_) FTIR DS obtained for a green alga PSI sample. In this case the PSI samples are monomeric.^25^

The FTIR DS in Figure 3 are obtained from PSI samples that are monomeric, dimeric, trimeric and tetrameric.

All the DS in Figure 3 share a high degree of similarity, with notably a difference band at 1745­(+)/1749(−) cm^–1^, and positive bands at ∼1495 and 1415 cm^–1^ being present in all the DS (shaded regions in Figure 3).

To more-fully demonstrate the similarity in the FTIR DS in Figure 3 the average of all 13 of the DS are presented in Figure 4. The spectra were min-max normalized in the 1800–1400 cm^–1^ region prior to averaging, with no accounting for differences in the number of flashes used to produce the final spectrum for each sample (this difference in number of flashes is typically small and is expected to have minimal impact on the averaged spectrum). The error bars in Figure 4 represents the standard error in all 13 of the DS. If the FTIR DS in Figure 3 exhibited considerable variability in both frequency and intensity then the error bars in the DS in Figure 4 should be considerably larger, and the difference bands more spread out and weaker. Given the intense bands and low noise level in the FTIR DS in Figure 4, we conclude a high degree of similarity in the 13 spectra in Figure 3, and the FTIR DS in Figure 4 can be considered a universal (A_1_^–^ – A_1_) FTIR DS that is applicable to any cyanobacterial or algal PSI sample, at least within the noise level set by the error bars.

**Figure 4:**
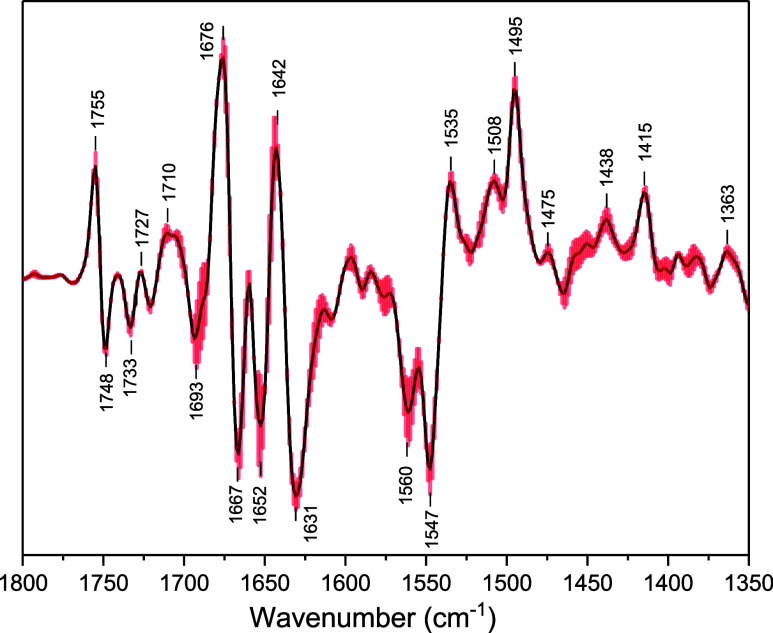
(A_1_^–^ – A_1_) FTIR DS obtained by averaging the FTIR DS in Figure 3. The error bars (*red shade*) indicate the standard error (plus/minus).

The small error bars in Figure 4 demonstrate high sensitivity, allowing an unambiguous resolution of even weak difference bands that were previously undetected or unreliably resolved.

## Discussion

The bands in the FTIR DS are usually discussed within the context of the molecular structural details of PhQ in the A_1_ binding site derived from crystal or cryo-em structures. Figure 5 shows two views of the A_1A_ binding site derived from the 2.5 Å PSI crystal structure from *TV*.^5^ The B-branch binding site is similar. Similar structures are obtained using plant^60^ or other cyanobacterial^22,61^ PSI crystal structures.

**Figure 5:**
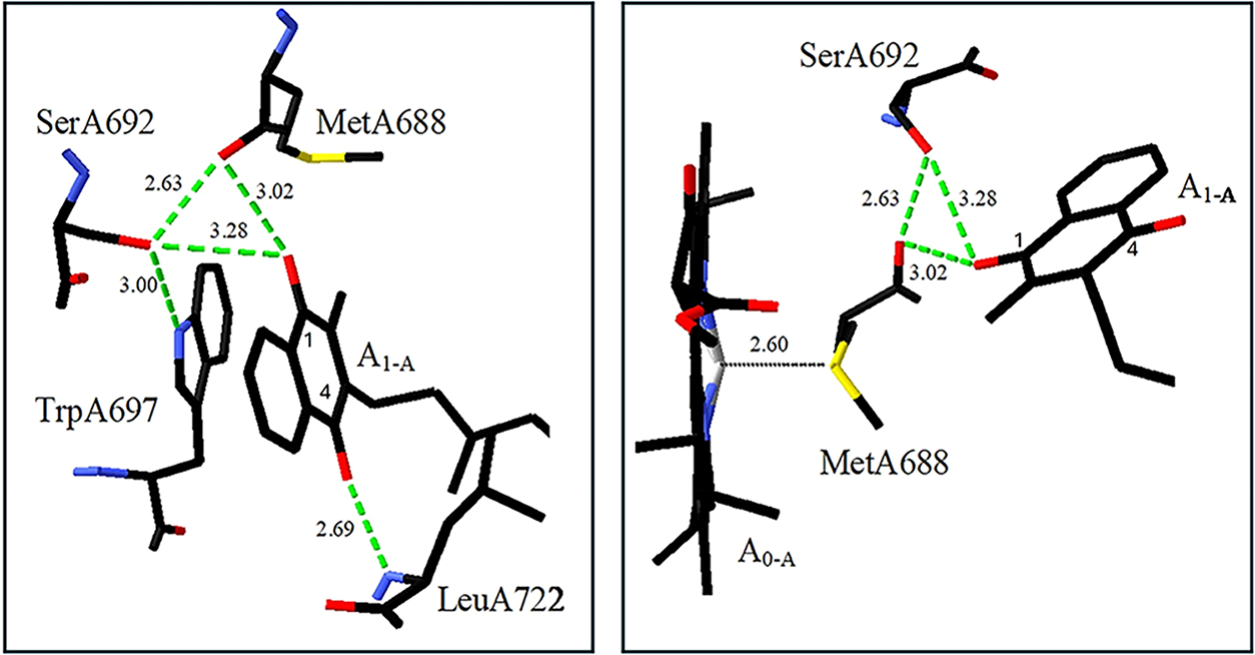
Two views of the structure of neutral PhQ in the A_1A_ binding site in PSI from *TV*. Possible H-bonds are shown dotted. Images are derived from PDB file 1JB0.^5^ Structure of PhQ in the A_1_ binding site on the B-branch is similar. Adapted from Figure 1 in ref 34.

The indole ring of TrpA697 is π-stacked with the PhQ ring plane. The hydroxyl oxygen of SerA692 is 2.63 Å from the backbone oxygen of MetA688, 3.00 Å from the indole nitrogen of TrpA697 and 3.28 Å from the carbonyl (C=O) oxygen of PhQ. From the geometry shown, SerA692 is most likely H-bonded to the backbone of MetA688. The C_4_=O group of PhQ is H-bonded to the backbone nitrogen of LeuA722. The sulfur atom of MetA688 provides a ligand to the A_0_ pigment. It is unclear if the C_1_=O of PhQ is H-bonded (as Figure 5 suggests). The available magnetic spectroscopic evidence indicates that it is not, or only very weakly, H bonded.^62^

### Neutral Phylloquinone Difference Bands

For neutral state quinones in solution, prominent FTIR bands due to C=O and C=C vibrations are found in the ∼1700–1600 cm^–1^ region.^63–69^ For neutral PhQ in solution, an intense IR absorption band is observed near 1662 cm^–1^.^66–70^ From density functional theory based normal mode vibrational frequency calculations it is shown that the 1662 cm^–1^ band is due primarily to an antisymmetric vibration of both C=O groups.^70,71^ For PhQ in an asymmetric H-bonding environment, such as that show in Figure 5, the C=O modes are expected to separate into distinct C_1_=O and C_4_=O vibrations (numbering is indicated in Figure 5).^35^

Upon anion formation the C=O and C=C groups of quinones undergo a large decrease in bond order, leading to C=O and C=C modes downshifting over 100 cm^–1^.^33,65,71^ This separation of neutral and anion bands of quinones simplifies to some degree spectral interpretation of bands in (A_1_^–^ – A_1_) FTIR DS. Despite this simplification difference bands in the neutral quinone region (∼1700–1600 cm^–1^) were difficult to assign because of multiple overlapping spectral features.

For the FTIR DS in Figure 4, there are two negative bands at 1652 and 1667 cm^–1^, and it had been concluded that it is the 1652 cm^–1^ band, and not the 1667 cm^–1^ band, that is due to a C_1_=O mode of neutral PhQ in the A_1A_ binding site. The reasons for this conclusion are as follows: PhQ in the A_1_ binding site is asymmetrically bound (Figure 5) and it is expected that the C=O modes will uncouple, and separate C_1_=O and C_4_=O modes should be observed.^35^ Both of these C=O modes may also be expected to occur at a lower frequency than PhQ in solvent, as this was what was found for the two C=O modes of neutral PhQ incorporated into the Q_A_ binding site in purple bacterial reaction centers.^67,72^ Since PhQ in THF exhibits a C=O band at 1662 cm^–1^,^66–69^ it was thought that a C=O mode of neutral PhQ in the A_1_ binding site must be lower than 1662 cm^–1^. Given this, a band at 1667 cm^–1^ is too high a frequency for a C=O mode of neutral PhQ in the A_1_ protein binding site. Therefore, the negative band at 1667 cm^–1^ in Figure 4 was thought *not* to be associated with a PhQ vibrational mode. In spite of this reasoning, a more recent study has provided strong evidence that the 1667 cm^–1^ band is in fact due to the C_1_=O mode of neutral PhQ in the A_1_ binding site.^51^

For the negative band at 1631 cm^–1^ in Figure 4 it is well established that this band is due to a C_4_=O mode of PhQ that is H-bonded to the backbone NH group of LeuA722. Thus, H-bonding downshifts the C_4_=O mode by 36 cm^–1^ compared to the C_1_=O mode. Table 1 lists these band assignments. All the DS in Figure 3 display negative bands at 1667 and 1631 cm^–1^ (note the low noise level associated with these bands in Figure 4) and hence the DS indicate a similar H-bonding environment around the C_1_=O and C_4_=O groups of PhQ in all PSI samples.

**Table 1:** Assignment of Bands in (A_1_^–^ – A_1_) FTIR DS

	frequency (cm^–1^)	assignment
1	1755(+)/1748(−)	13^3^ ester C=O of A_0_
2	1710(+)/1693(−)	13^1^ keto C=O of A_0_
3	1693(−)	amide I (ground state?)
4	1677(+)	amide I (P700^+^A_1_^–^ state)
5	1667(−)	C_1_=O of neutral PhQ
6	1652(−)	amide I (ground state)
7	1642(+)	amide I (P700^+^A_1_^–^ state)
8	1631(−)	C_4_=O of neutral PhQ
9	1560(−)	amide II (ground state)
10	1547(−)	amide II (ground state)
11	1535(+)	amide II (P700^+^A_1_^–^ state)
12	1508(+)	$C\overset{\cdot \cdot \cdot }{-}C\;\mathrm{of}\;{\mathrm{PhQ}}^{-}$
13	1495(+)	$C_{1}\overset{\cdot \cdot \cdot }{-}O\;\mathrm{of}\;{\mathrm{PhQ}}^{-}$
14	1475(+)	δ(CH_3_) of PhQ^–^
15	1438(+)	Not assigned
16	1415(+)	$C_{4}\overset{\cdot \cdot \cdot }{-}O\;\mathrm{of}\;{\mathrm{PhQ}}^{-}$
17	1363(+)	Not assigned

For neutral PhQ in solution, C=C modes are found near 1619 cm^–1^.^70,71^ Such bands are at least a factor of 8 lower in intensity than the C=O modes, however.^70,71^ With this in mind, we expect that bands due to C=C modes of neutral PhQ will be weak and difficult to observe in the FTIR DS in Figure 4. A negative band that is barely above the noise level is observed near 1610 cm^–1^ in Figure 4 and could potentially be assigned to a PhQ C=C mode.

### Amide I and II Protein Absorption Bands

Negative/positive bands in FTIR DS are associated with the neutral/anion state, respectively. Since PhQ C=O and C=C modes downshift more than 100 cm^–1^ upon anion formation,^71^ positive bands in the neutral region cannot be associated with modes of PhQ^–^. In Figure 4 there are two very clear positive bands at 1677 and 1642 cm^–1^, and both are likely due to amide I protein absorption bands that are shifted to these frequencies upon P700^+^A_1_^–^ formation. The frequencies for these amide I bands in the neutral state are less clear. It is probable that the negative band at 1652 cm^–1^ downshifts to 1642 cm^–1^ upon P700^+^A_1_^–^ formation. Although it cannot be ruled out that it upshifts to 1677 cm^–1^ upon P700^+^A_1_^–^ formation. There has also been some experimental support for the idea that the negative 1693 cm^–1^ band is due to an amide I mode that downshifts to 1677 cm^–1^ upon P700^+^A_1_^–^ formation. An amide I mode at 1693 cm^–1^ suggests an antiparallel β-sheet type secondary structure, or potentially a turns structure but not an α-helical structure.^73^ Another hypothesis as to the origin of the 1693 cm^–1^ band is given below.

Since there are two amide I difference bands in Figure 4, the expectation would be that there should also be two amide II difference bands. Amide II bands occur around 1550 cm^–1^.^73^ In Figure 4 two negative bands are observed at 1560 and 1547 cm^–1^. One suggestion could be that the 1547 cm^–1^ band downshifts to 1535 cm^–1^, while the 1560 cm^–1^ band upshifts to near 1575 cm^–1^.

An amide I (or II) band-shift may suggest an alteration in protein secondary structure upon radical pair formation, or may be associated with an electrochromic shift or mode structure modification caused by the electric field set up between P700^+^ and A_1_^–^. The impacted amide I modes may be due to peptide groups near the P700 pigments or the A_1_ pigment.

Another hypothesis is that difference features in the neutral region could be due to amino acid side-chain vibrations, rather than peptide backbone vibrations. Gln and Asn C=O groups could give rise to a vibrations near 1650–1670 cm^–1^.^73^ However, such amino acid vibrations may be expected to give a weak band in the DS, as C=O groups of Asn and Gln residues are distant from the charge centers. The 1677 cm^–1^ band also downshifts ∼40 cm^–1^ upon ^13^C labeling.^34^ This observation argues against this band being due to a C=O group of Asn and Gln, as ester or keto C=O vibrations to expected to downshift in excess of 42 cm^–1^ upon ^13^C labeling.^34^ A ^13^C-induced downshift of ∼40 cm^–1^ (and not 42–44 cm^–1^) is more in line with that expected for an amide I vibration.^51^

In a previous study at lower sensitivity, it was suggested the 1667(−) cm^–1^ band is found at a lower frequency in monomeric PSI from *S6803*.^34^ The data presented here for monomeric PSI from *S6803* indicate no such change. The monomeric PSI samples from *S6803* used previously were from a mutant that lacked PSII (the genes for PSII proteins had been deleted). The current sample had PSII separated from PSI via a His-tag and Ni-NTA column chromatography, allowing the easy resolution of a green band due to monomeric PSI in a sucrose density gradient. It is possible the spectral differences arise from the different source of PSI samples. It is also possible there are artifacts in the previous data due to the use of lower sensitivity instrumentation and sample preparation methods. Most likely some combination of both. In any case the DS in Figure 3 clearly indicate a 1667(−)/1677­(+) cm^–1^ difference band for monomeric PSI from *S6803*.

### Spectral Signatures Associated with the A_0_ Pigment in (A_1_^–^ – A_1_) FTIR DS

The A_0_ pigment is a Chl *a* molecule, the numbering scheme for which is indicated in Figure 1B. Previously it was suggested that the 1748(−)/1755­(+) cm^–1^ difference band is due to a 13^3^ ester C=O vibration of the A_0_ pigment.^34^ This latter idea comes from a study of a site directed mutant where MetA688 is changed to Leu. MetA688 is the ligand to the A_0_ pigment (Figure 5). In the MetA688 to Leu mutation the 1748(−)/1755­(+) cm^–1^ difference band disappears.^74^ However, the band is still observed in the corresponding mutation on the B-branch.^74^ The latter observation would be expected since ET is down the A-branch at 77 K. So, the suggestion is that the 13^3^ ester C=O vibration of A_0A_ upshifts 7 cm^–1^ upon A_1A_^–^ formation. This upshift is most likely an electrochromic shift of the 13^3^ ester C=O group of A_0_, but there might also be some structural alteration in the A_0_ pigment in response to radical pair formation. Such contributions of distant pigments to FTIR DS are well-known. For example, bands due to bacteriopheophytin are known to contribute to (Q_A_^–^ – Q_A_) FTIR DS obtained using purple bacterial reaction centers.^75,76^

If the 1748(−)/1755­(+) cm^–1^ difference band is due to a 13^3^ ester C=O vibration of A_0_ then one might also expect to observe bands in the DS due to the 13^1^ keto C=O vibration of A_0_. One suggestion is that the 1693(−)/1710­(+) cm^–1^ difference band is due to a 13^1^ keto C=O vibration of A_0_. Keto C=O vibrations are well-known to occur near 1693 cm^–1^, and this assignment is consistent with observed ^13^C and ^2^H isotope induced downshifts.^31,34^ However, the 13^1^-keto C=O of A_0_ is H-bonded to TyrA696,^34^ which might suggest a lower frequency for this mode. Currently, the origin of the 1693(−) cm^–1^ difference band has yet to be verified. An alternative assignment for the 1693(−) cm^–1^ band was indicated above. Table 1 list some of the discussed band assignments.

### Phylloquinone Anion Bands

In the anion spectral region (1550–1400 cm^–1^) the two most prominent features are the positive bands at 1495 and 1415 cm^–1^. It is well established that these bands are due to $C_{1}\overset{\cdot \cdot \cdot }{-}O$ and $C_{4}\overset{\cdot \cdot \cdot }{-}O$ vibrations of PhQ^–^, respectively, the latter being more mixed with C=C stretching and CH bending modes.^33^ Thus, the H-bond environment around the $C_{4}\overset{\cdot \cdot \cdot }{-}O$ group causes a ∼80 cm^–1^ downshift in the $C_{4}\overset{\cdot \cdot \cdot }{-}O$ mode compared to the $C_{1}\overset{\cdot \cdot \cdot }{-}O$ mode. Of course, as mentioned, what we are calling a $C_{4}\overset{\cdot \cdot \cdot }{-}O$ mode is in fact a more complicated vibration containing increased contributions from other molecular groups of the quinone. Interestingly, for the neutral quinone the C_1_=O and C_4_=O groups are separated by only 36 cm^–1^. So, there may be some alteration of PhQ or other molecular groups upon radical formation that increase the strength of H-bonding to the C_4_=O group.

In the anion region, $C\overset{\cdot \cdot \cdot }{-}C$ modes of the quinone ring often appear at higher frequencies than the $C\overset{\cdot \cdot \cdot }{-}O$ modes,^65^ and it has been suggested that the band at 1508­(+) cm^–1^ in the DS in Figure 4 is due to a $C\overset{\cdot \cdot \cdot }{-}C$ mode of PhQ^–^ (Table 1).^77^ In some strains the frequency is slightly higher, and there is often spectral overlap with other bands (Figure S2–S5).^31^ However, a band is very clearly observed at 1508­(+) cm^–1^ in Figure 4 and is assigned to a $C\overset{\cdot \cdot \cdot }{-}C$ mode of PhQ^–^ (Table 1).

Positive bands are also clearly observed at 1475 and 1438 cm^–1^. The band at 1475 cm^–1^ was assigned to a CH bending vibration of the methyl group at the C_2_ position of PhQ (in the anion state).^51,78^ The band at 1438 cm^–1^ has not been assigned. Previously, the low intensity of the 1475 and 1438 cm^–1^ bands, and even the 1508 cm^–1^ band, made their observation difficult, but they are now clearly observed in the DS in Figure 4.

In the past (A_1_^–^ – A_1_) FTIR DS were generally not presented below 1400 cm^–1^. Data is presented down to 1350 cm^–1^ in Figure 4, however, and a positive band is observed at 1363 cm^–1^. The origin of this 1363 cm^–1^ band is not clear at present.

### PSI from Other Strains

(A_1_^–^ – A_1_) FTIR DS have never been presented for *CT7203* (WL or FRL), *FT7521* (WL or FRL) and *CTS821* PSI samples (cells from *CTS821* are not capable of growth under FRL^79^). Figures S3 and S4 show expanded views and comparison of the DS for WL and FRL PSI samples, respectively. There are some small differences in the DS, mostly in the 1660–1630 cm^–1^ region, where the sensitivity is lowest. There are also differences in the 1560 and 1547 cm^–1^ bands. Again, differences in this region are expected due to sample heating artifacts.

It is noteworthy that the (A_1_^–^ – A_1_) FTIR DS are similar for the FRL- and WL-PSI samples from *CT7203*. This is interesting because the (P700^+^ – P700) FTIR DS are very different for the FRL and WL-PSI samples from *CT7203* (*not shown*). This topic will be addressed in a future manuscript. For now, we suggest that the similarity in structure that extends from A_0_ to A_1_ (as established through the (A_1_^–^ – A_1_) FTIR DS) does not extend to P700, at least for the WL- and FRL PSI samples for *CT7203*.

Several cyanobacteria encode two distinct sets of PSI genes: one expressed under normal WL conditions (typical of full-spectrum sunlight), and another expressed only under FRL (700–800 nm), as part of an acclimation response known as FaRLiP (far-red light photoacclimation).^79,80^ Phylogenetic analysis of the PsaL subunit indicates that the FRL–induced PSI adopts the trimeric organization, whereas the WL–expressed PSI encodes a divergent PsaL that drives tetramer formation.^81^ Structural studies have shown that this tetramer is best described as a “dimer-of-dimers” with its relative abundance depending on both isolation conditions and protein concentration.^82^ Although the structure of *CT7203* WL form of PSI has not yet been solved, it is very likely to adopt a tetrameric organization similar to that observed for *CTS821*. Atomic force microscopy (AFM) and native PAGE experiments support this.^47^ However, upon isolation of PSI samples from WL-grown cells from *CT7203* it appears that the tetrameric arrangement is degraded to a dimeric form under the conditions used.^47^

By including these three types of PSI from two related *Chroococcidiopsidales* in our FTIR analysis, and in addition including the tetrameric PSI strain from *LO*, we demonstrate that the tetrameric form of PSI is consistently associated with the same set of FTIR spectral signatures as the trimeric or dimeric form (Figure S3).

Figure S5 shows an expanded view of the (A_1_^–^ – A_1_) time-resolved FTIR DS for PSI from the green alga *Chlamydomonas reinhardtii* (*CR*). The PSI oligomeric state in *CR* depends on growth conditions and handling procedures. In most reports—and in the samples analyzed here—PSI from *CR* is monomeric.^83,84^ However, there is new evidence that the PSI from *CR* may also exist in a dimeric form.^25^ However, it is notable that the majority of bands in Figure S5 are shared with those observed in spectra for the other strains, indicating that the (A_1_^–^ – A_1_) FTIR DS is invariant across PSI oligomeric states (monomer, dimer, trimer, and tetramer).

## Conclusions

The averaging of 13 (A_1_^–^ – A_1_) FTIR DS from many types of PSI samples results in a highly resolved (A_1_ ® – A_1_) FTIR DS. This indicates considerable similarity in the 13 underlying DS. The resulting averaged (A_1_^–^ – A_1_) FTIR DS can therefore be considered as representative of that expected for PSI from all cyanobacteria and green algae. (A_1_^–^ – A_1_) FTIR DS have previously been obtained for a few cyanobacterial PSI strains (*S6803*, *S7002*, *TV*). The work reported here extends this to eight phylogenetically diverse strains and a green algae.

The composite (A_1_^–^ – A_1_) FTIR DS is independent of the oligomeric nature of the PSI samples (monomer, dimer, trimer or tetramer). It is also independent whether the PSI samples came from cells grown under white light or far-red light.

The similarity in the DS of all strains provides compelling evidence indicating the robustness of the data collection procedures.

With a solid estimate of the experimental variability, it is shown that even weak bands can be resolved, particularly bands at 1508, 1475, 1438, and 1363 cm^–1^.

Most of the bands in the (A_1_^–^ – A_1_) FTIR DS are dependent on the details of the environment surrounding the pigments in both the A_1_ and the A_0_ binding sites. The fact that the DS are similar for all strains suggests that most, if not all, of the structural details between A_0_ and A_1_ are preserved in all strains considered here, in both neutral and radical states.

## Supplementary Material



## Footnotes

1 Abbreviations: C=O, carbonyl; *CR*, *Chlamydomonas reinhardtii*; Chl *a*, chlorophyll *a*; *CT7203, Chroococcidiopsis thermalis* PCC 7203; *CTS821*, *Chroococcidiopsis* sp. TS-821; DS, difference spectra/spectrum/spectroscopy; ET, electron transfer; FRL, far red light; *FT7521*, *Fischerella thermalis* PCC 7521; FTIR, Fourier transform infrared; H-bond, hydrogen bond; *LO*, *Leptolyngbya ohadii*; PhQ, phylloquinone (2-methyl-3-phytyl-1,4-naphthoqiuinone); PSI, photosystem I; PSII, photosystem II; RC, reaction center; *S7942*, *Synechococcus elongatus* sp. PCC 7942; *S6803*, *Synechocystis* sp. PCC 6803; *TV*, *Thermosynechococcus vestitus*; TRSS, time-resolved step-scan; WL, white light; WT, wild type.

## Notes

Expanded views of the difference spectra outlined in Figure 3; expanded view of difference spectra for PSI from S6803, TV, and S7002 (Figure S1), for PSI from CTS821, WL-PSI from FT7521, and CT7203 (Figure S3), for FRL-PSI from FT7521 and CT7203 (Figure S4), and for PSI from CR (Figure S5); and spectrum of a heating artifact found upon laser excitation of PSI samples at 77 K (Figure S2) (PDF)

## References

[1] Walker, D. Energy, Plants and Man, 2nd ed.; Oxygraphics: Brighton, East Sussex: Mill Valley, CA, 1993.

[2] Barber, J. The Photosystems: Structure, Function, and Molecular Biology; Elsevier Science Publishers: Amsterdam, NY, 1992; Vol. 11.

[3] Golbeck, J. ; Bryant, D. Photosystem I. In Current Topics in Bioenergetics; Academic Press: New York, 1991; Vol. 16, pp 83–175.

[4] Webber A. N., Lubitz W. (2001). P700: the primary electron donor of photosystem I. Biochim. Biophys. Acta, Bioenerg..

[5] Jordan P., Fromme P., Witt H. T., Klukas O., Saenger W., Krauss N. (2001). Three-dimensional structure of cyanobacterial photosystem
I at 2.5 Å resolution. Nature.

[6] Nakamura A., Akai M., Yoshida E., Taki T., Watanabe T. (2003). Reversed-phase
HPLC determination of chlorophyll a’ and phylloquinone in Photosystem
I of oxygenic photosynthetic organisms. Universal existence of one
chlorophyll a’ molecule in Photosystem I. Eur. J. Biochem..

[7] Holzwarth A. R., Muller M. G., Niklas J., Lubitz W. (2006). Ultrafast transient
absorption studies on photosystem I reaction centers from Chlamydomonas
reinhardtii. 2: mutations near the P700 reaction center chlorophylls
provide new insight into the nature of the primary electron donor. Biophys. J..

[8] Cherepanov D. A., Shelaev I. V., Gostev F. E., Petrova A., Aybush A. V., Nadtochenko V. A., Xu W., Golbeck J. H., Semenov A. Y. (2021). Primary
charge separation within the structurally symmetric tetrameric Chl2APAPBChl2B
chlorophyll exciplex in photosystem I. J. Photochem.
Photobiol., B.

[9] Nürnberg D. J., Morton J., Santabarbara S., Telfer A., Joliot P., Antonaru L. A., Ruban A. V., Cardona T., Krausz E., Boussac A., Fantuzzi A., Rutherford A. W. (2018). Photochemistry
beyond the red limit in chlorophyll *f*–containing
photosystems. Science.

[10] Hastings G., Makita H., Agarwala N., Rohani L., Shen G., Bryant D. A. (2019). Fourier transform visible and infrared difference spectroscopy
for the study of P700 in photosystem I from Fischerella thermalis
PCC 7521 cells grown under white light and far-red light: Evidence
that the A_–1_ cofactor is chlorophyll *f*. Biochim. Biophys. Acta, Bioenerg..

[11] Chen, M. Chlorophylls *d* and *f*: Synthesis, occurrence, light-harvesting, and pigment organization in chlorophyll-binding protein complexes. In Advances in botanical research; Grimm, B. , Ed.; Academic Press, 2019; Vol. 90, pp 121–139.

[12] Brettel K. (1997). Electron transfer
and arrangement of the redox cofactors in photosystem I. Biochim. Biophys. Acta, Bioenerg..

[13] Golbeck J. H. (1992). Structure
and Function of Photosystem I. Annu. Rev. Plant
Physiol. Plant Mol. Biol..

[14] Hastings G., Hoshina S., Webber A. N., Blankenship R. E. (1995). Universality
of Energy and Electron-Transfer Processes in Photosystem-I. Biochemistry.

[15] Hastings G., Kleinherenbrink F., Lin S., McHugh T., Blankenship R. (1994). Observation
of the reduction and reoxidation of the primary electron acceptor
in photosystem I. Biochemistry.

[16] Brettel K., Leibl W. (2001). Electron transfer in photosystem I. Biochim.
Biophys. Acta, Bioenerg..

[17] Mäusle S. M., Agarwala N., Eichmann V. G., Dau H., Nürnberg D. J., Hastings G. (2024). Nanosecond time-resolved infrared spectroscopy for
the study of electron transfer in photosystem I. Photosynth. Res..

[18] Agalarov R., Brettel K. (2003). Temperature dependence of biphasic forward electron
transfer from the phylloquinone(s) A_1_ in photosystem I:
only the slower phase is activated. Biochim.
Biophys. Acta, Bioenerg..

[19] Makita H., Zhao N., Hastings G. (2015). Time-resolved visible and infrared
difference spectroscopy for the study of photosystem I with different
quinones incorporated into the A_1_ binding site. Biochim. Biophys. Acta, Bioenerg..

[20] Schlodder E., Falkenberg K., Gergeleit M., Brettel K. (1998). Temperature dependence
of forward and reverse electron transfer from A_1_
^–^, the reduced secondary electron acceptor in photosystem I. Biochemistry.

[21] Makita H., Hastings G. (2015). Directionality of electron transfer in cyanobacterial
photosystem I at 298 and 77 K. Febs Lett..

[22] Keable S. M., Kolsch A., Simon P. S., Dasgupta M., Chatterjee R., Subramanian S. K., Hussein R., Ibrahim M., Kim I. S., Bogacz I., Makita H., Pham C. C., Fuller F. D., Gul S., Paley D., Lassalle L., Sutherlin K. D., Bhowmick A., Moriarty N. W., Young I. D., Blaschke J. P., de Lichtenberg C., Chernev P., Cheah M. H., Park S., Park G., Kim J., Lee S. J., Park J., Tono K., Owada S., Hunter M. S., Batyuk A., Oggenfuss R., Sander M., Zerdane S., Ozerov D., Nass K., Lemke H., Mankowsky R., Brewster A. S., Messinger J., Sauter N. K., Yachandra V. K., Yano J., Zouni A., Kern J. (2021). Room temperature XFEL
crystallography reveals asymmetry in the vicinity of the two phylloquinones
in photosystem I. Sci. Rep..

[23] Santabarbara S., Casazza A. P. (2019). Kinetics and Energetics of Phylloquinone Reduction
in Photosystem I: Insight From Modeling of the Site Directed Mutants. Front. Plant Sci..

[24] Mitsuhashi K., Tamura H., Saito K., Ishikita H. (2021). Nature of Asymmetric
Electron Transfer in the Symmetric Pathways of Photosystem I. J. Phys. Chem. B.

[25] Caspy I., Schwartz T., Bayro-Kaiser V., Fadeeva M., Kessel A., Ben-Tal N., Nelson N. (2021). Dimeric and high-resolution structures
of Chlamydomonas Photosystem I from a temperature-sensitive Photosystem
II mutant. Commun. Biol..

[26] Byrdin M., Santabarbara S., Gu F. F., Fairclough W. V., Heathcote P., Redding K., Rappaport F. (2006). Assignment
of a kinetic component to electron transfer between iron-sulfur clusters
F_X_ and F_A/B_ of Photosystem I. Biochim. Biophys. Acta, Bioenerg..

[27] Makita H., Hastings G. (2016). Modeling electron transfer in photosystem I. Biochim. Biophys. Acta, Bioenerg..

[28] van
der Est A. (2001). Light-induced spin polarization in type I photosynthetic
reaction centres. Biochim. Biophys. Acta, Bioenerg..

[29] van der Est, A. Electron Transfer Involving Phylloquinone in Photosystem I. In Photosystem I: The Light Driven Plastocyanin:Ferredoxin Oxidoreductase; Golbeck, J. , Ed.; Springer: Dordrecht, 2006; pp 387–411.

[30] Hastings G. (2001). Time-resolved
step-scan Fourier transform infrared and visible absorption difference
spectroscopy for the study of photosystem I. Appl. Spectrosc..

[31] Hastings, G. FTIR Studies of the Intermediate Electron Acceptor A_1_ . In Photosystem I: The Light Driven Plastocyanin:Ferredoxin Oxidoreductase; Golbeck, J. , Ed.; Springer: Dordrecht, 2006; pp 301–318.

[32] Mezzetti, A. Photobiological systems studied by time-resolved infrared spectroscopy (2015–2018). Photochemistry, Vol 47, Albini, A. ; Protti, S. , Eds. 2020; Vol. 47, pp 159–195.

[33] Hastings G. (2015). Vibrational
spectroscopy of photosystem I. Biochim. Biophys.
Acta, Bioenerg..

[34] Sivakumar V., Wang R., Hastings G. (2005). A_1_ reduction in intact
cyanobacterial photosystem I particles studied by time-resolved step-scan
fourier transform infrared difference spectroscopy and isotope labeling. Biochemistry.

[35] Bandaranayake K. M. P., Wang R., Hastings G. (2006). Modification of the Phylloquinone
in the A_1_ Binding Site in Photosystem I Studied Using Time-Resolved
FTIR Difference Spectroscopy and Density Functional Theory. Biochemistry.

[36] Bandaranayake K. M. P., Wang R., Johnson T. W., Hastings G. (2006). Time-resolved FTIR
difference spectroscopy for the study of photosystem I particles with
plastoquinone-9 occupying the A_1_ binding site. Biochemistry.

[37] Johnson T. W., Shen G., Zybailov B., Kolling D., Reategui R., Beauparlant S., Vassiliev I. R., Bryant D. A., Jones A. D., Golbeck J. H., Chitnis P. R. (2000). Recruitment of a foreign quinone
into the A1 site of photosystem I. I. Genetic and physiological characterization
of phylloquinone biosynthetic pathway mutants in Synechocystis sp.
pcc 6803. J. Biol. Chem..

[38] Bricker T. M., Morvant J., Masri N., Sutton H. M., Frankel L. K. (1998). Isolation
of a highly active Photosystem II preparation from Synechocystis 6803
using a histidine-tagged mutant of CP 47. Biochim.
Biophys. Acta, Bioenerg..

[39] Kashino Y., Lauber W. M., Carroll J. A., Wang Q., Whitmarsh J., Satoh K., Pakrasi H. B. (2002). Proteomic Analysis of a Highly Active
Photosystem II Preparation from the Cyanobacterium Synechocystis sp.
PCC 6803 Reveals the Presence of Novel Polypeptides. Biochemistry.

[40] Sun J., Ke A., Jin P., Chitnis V. P., Chitnis P. R. (1998). Isolation and functional
study of photosystem I subunits in the cyanobacterium Synechocystis
sp. PCC 6803. Methods Enzymol..

[41] Kurashov V., Milanovsky G., Luo L., Martin A., Semenov A. Y., Savikhin S., Cherepanov D. A., Golbeck J. H., Xu W. (2021). Conserved
residue PsaB-Trp673 is essential for high-efficiency electron transfer
between the phylloquinones and the iron-sulfur clusters in Photosystem
I. Photosynth. Res..

[42] Johnson T. W., Shen G., Zybailov B., Kolling D., Reategui R., Beauparlant S., Vassiliev I. R., Bryant D. A., Jones A. D., Golbeck J. H., Chitnis P. R. (2000). Recruitment of a Foreign Quinone
into the A1 Site of Photosystem I: I. Genetic and Physiological Characterization
of Phylloquinone Biosynthetic Pathway Mutants in *Synechocystis* sp. PCC 6803. J. Biol. Chem..

[43] Hastings G., Bandaranayake K. M. P., Carrion E. (2008). Time-resolved FTIR difference spectroscopy
in combination with specific isotope labeling for the study of A_1_, the secondary electron acceptor in photosystem 1. Biophys. J..

[44] Shen G., Zhao J., Reimer S. K., Antonkine M. L., Cai Q., Weiland S. M., Golbeck J. H., Bryant D. A. (2002). Assembly of photosystem
I. I. Inactivation of the rubA gene encoding a membrane-associated
rubredoxin in the cyanobacterium Synechococcus sp. PCC 7002 causes
a loss of photosystem I activity. J. Biol. Chem..

[45] Hastings G., Reed L. J., Lin S., Blankenship R. E. (1995). Excited
state dynamics in photosystem I: Effects of detergent and excitation
wavelength. Biophys. J..

[46] Rippka R., Deruelles J., Waterbury J. B., Herdman M., Stanier R. Y. (1979). Generic
Assignments, Strain Histories and Properties of Pure Cultures of Cyanobacteria. Microbiology.

[47] MacGregor-Chatwin C., Nürnberg D. J., Jackson P. J., Vasilev C., Hitchcock A., Ho M.-Y., Shen G., Gisriel C. J., Wood W. H. J., Mahbub M., Selinger V. M., Johnson M. P., Dickman M. J., Rutherford A. W., Bryant D. A., Hunter C. N. (2022). Changes in supramolecular
organization of cyanobacterial thylakoid membrane complexes in response
to far-red light photoacclimation. Sci. Adv..

[48] Semchonok D. A., Mondal J., Cooper C. J., Schlum K., Li M., Amin M., Sorzano C. O. S., Ramírez-Aportela E., Kastritis P. L., Boekema E. J., Guskov A., Bruce B. D. (2022). Cryo-EM
structure of a tetrameric photosystem I from Chroococcidiopsis TS-821,
a thermophilic, unicellular, non-heterocyst-forming cyanobacterium. Plant Commun..

[49] Wang R., Sivakumar V., Li Y., Redding K., Hastings G. (2003). Mutation Induced
Modulation of Hydrogen Bonding to P700 Studied Using FTIR Difference
Spectroscopy. Biochemistry.

[50] Niedzwiedzki D. M., Magdaong N. C. M., Su X., Adir N., Keren N., Liu H. (2023). Mass spectrometry and spectroscopic characterization of a tetrameric
photosystem I supercomplex from *Leptolyngbya ohadii*, a desiccation-tolerant cyanobacterium. Biochim.
Biophys. Acta, Bioenerg..

[51] Makita H., Hastings G. (2020). Time-resolved FTIR difference spectroscopy for the
study of quinones in the A_1_ binding site in photosystem
I: Identification of neutral state quinone bands. Biochim. Biophys. Acta, Bioenerg..

[52] Breton, J. FTIR Studies of the Primary Electron Donor, P700. In Photosystem I: The Light Driven Plastocyanin:Ferredoxin Oxidoreductase; Golbeck, J. , Ed.; Springer: Dordrecht, 2006; pp 271–289.

[53] Agarwala N., Makita H., Luo L., Xu W., Hastings G. (2020). Reversible
inhibition and reactivation of electron transfer in photosystem I. Photosynth. Res..

[55] Makita H., Rohani L., Zhao N., Hastings G. (2017). Quinones in the A_1_ binding site in photosystem I studied using time-resolved
FTIR difference spectroscopy. Biochim. Biophys.
Acta, Bioenerg..

[56] Makita H., Hastings G. (2019). Time-Resolved Step-Scan FTIR Difference Spectroscopy
for the Study of the P700 Triplet State in Photosystem I. Front. Sci. Technol. Eng. Math..

[57] Rödig C., Siebert F. (1999). Errors and artifacts in time-resolved step-scan FT-IR
spectroscopy. Appl. Spectrosc..

[58] Mezzetti A., Spezia R. (2008). Time-resolved step scan FTIR spectroscopy and DFT investigation
on triplet formation in peridinin-chlorophyll-a-protein from Amphidinium
carterae at low temperature. J. Spectrosc..

[59] Gisriel C., Shen G., Kurashov V., Ho M.-Y., Zhang S., Williams D., Golbeck J. H., Fromme P., Bryant D. A. (2020). The structure
of Photosystem I acclimated to far-red light illuminates an ecologically
important acclimation process in photosynthesis. Sci. Adv..

[60] Mazor Y., Borovikova A., Nelson N. (2015). The structure of plant photosystem
I super-complex at 2.8 Å resolution. eLife.

[61] Mazor Y., Nataf D., Toporik H., Nelson N. (2014). Crystal structures
of virus-like photosystem I complexes from the mesophilic cyanobacterium
Synechocystis PCC 6803. eLife.

[62] Srinivasan N., Golbeck J. H. (2009). Protein-cofactor interactions in bioenergetic complexes:
The role of the A_1A_ and A_1B_ phylloquinones in
Photosystem I. Biochim. Biophys. Acta, Bioenerg..

[63] Bauscher M., Mantele W. (1992). Electrochemical and Infrared-Spectroscopic Characterization
of Redox Reactions of p-Quinones. J. Phys. Chem.
A.

[64] Bauscher M., Nabedryk E., Bagley K., Breton J., Mäntele W. (1990). Investigation
of models for photosynthetic electron acceptors: Infrared spectroelectrochemistry
of ubiquinone and its anions. Febs Lett..

[65] Breton J., Nabedryk E. (1996). Protein-quinone interactions in the bacterial photosynthetic
reaction center: Light-induced FTIR difference spectroscopy of the
quinone vibrations. Biochim. Biophys. Acta,
Bioenerg..

[66] Hastings G., Sivakumar V. (2001). A Fourier transform infrared absorption difference
spectrum associated with the reduction of A_1_ in photosystem
I: are both phylloquinones involved in electron transfer?. Biochemistry.

[67] Breton J., Burie J. R., Berthomieu C., Berger G., Nabedryk E. (1994). The binding
sites of quinones in photosynthetic bacterial reaction centers investigated
by light-induced FTIR difference spectroscopy: assignment of the Q_A_ vibrations in R*hodobacter sphaeroides* using ^18^O- or ^13^C-labeled ubiquinone and vitamin K_1_. Biochemistry.

[68] Agarwala N., Rohani L., Hastings G. (2022). Experimental and calculated infrared
spectra of disubstituted naphthoquinones. Spectrochim.
Acta, Part A.

[69] Lim Z. H., Chng E. L. K., Hui Y., Webster R. D. (2013). The Hydrogen-Bonded
Dianion of Vitamin K1 Produced in Aqueous–Organic Solutions
Exists in Equilibrium with Its Hydrogen-Bonded Semiquinone Anion Radical. J. Phys. Chem. B.

[70] Agarwala N., Ranke D., Rohani, Hastings G. (2019). Calculated and Experimental Infrared
Spectra of Substituted Naphthoquinones. Front.
Sci. Technol. Eng. Math..

[71] Bandaranayake K., Sivakumar V., Wang R., Hastings G. (2006). Modeling The A_1_ Binding Site In Photosystem: I. Density Functional Theory
For The Calculation Of “Anion – Neutral” FTIR
Difference Spectra of Phylloquinone. Vib. Spectrosc..

[72] Breton J., Burie J. R., Boullais C., Berger G., Nabedryk E. (1994). Binding sites
of quinones in photosynthetic bacterial reaction centers investigated
by light-induced FTIR difference spectroscopy: binding of chainless
symmetrical quinones to the Q_A_ site of *Rhodobacter
sphaeroides*. Biochemistry.

[73] Barth, A. , Infrared spectroscopy of proteins. Biochim. Biophys. Acta, Bioenerg. 2007, 1767. 10.1016/j.bbabio.2007.06.004. 17692815

[74] Sivakumar, V. ; Wang, R. ; Johnson, T. ; Hastings, G. A_1_ Reduction in Intact Cyanobacterial Photosystem I Studied Using Time-resolved Step-scan Fourier Transform Infra-red Difference Spectroscopy In Combination With Site Directed Mutagenesis and Quinone Exchange Experiments. In Photosynthesis: Fundamental Aspects to Global Perspectives; van der Est, A. ; Bruce, D. , Eds.; Alliance Communications Group: Lawrence, KS, 2005; pp 59–60.

[75] Breton J., Bibikova M., Oesterhelt D., Nabedryk E. (1999). Conformational heterogeneity
of the bacteriopheophytin electron acceptor H_A_ in reaction
centers from *Rhodopseudomonas viridis* revealed by
Fourier transform infrared spectroscopy and site-directed mutagenesis. Biochemistry.

[76] Breton J., Nabedryk E., Allen J. P., Williams J. C. (1997). Electrostatic Influence
of QA Reduction on the IR Vibrational Mode of the 10a-Ester CO of
HA Demonstrated by Mutations at Residues Glu L104 and Trp L100 in
Reaction Centers from Rhodobacter sphaeroides. Biochemistry.

[77] Agarwala N., Makita H., Hastings G. (2023). Time-resolved FTIR difference spectroscopy
for the study of photosystem I with high potential naphthoquinones
incorporated into the A_1_ binding site. Biochim. Biophys. Acta, Bioenerg..

[78] Rohani L., Makita H., Levitz A., Henary M., Hastings G. (2019). Calculated
vibrational properties of semiquinones in the A_1_ binding
site in photosystem I. Biochim. Biophys. Acta.
Bioenerg..

[79] Antonaru L. A., Rad-Menéndez C., Mbedi S., Sparmann S., Pope M., Oliver T., Wu S., Green D. H., Gugger M., Nürnberg D. J. (2025). Evolution of far-red light photoacclimation in cyanobacteria. Curr. Biol..

[80] Gan F., Zhang S., Rockwell N. C., Martin S. S., Lagarias J. C., Bryant D. A. (2014). Extensive remodeling of a cyanobacterial photosynthetic
apparatus in far-red light. Science.

[81] Li M., Calteau A., Semchonok D. A., Witt T. A., Nguyen J. T., Sassoon N., Boekema E. J., Whitelegge J., Gugger M., Bruce B. D. (2019). Physiological and evolutionary implications
of tetrameric photosystem I in cyanobacteria. Nat. Plants.

[82] Semchonok D. A., Li M., Bruce B. D., Oostergetel G. T., Boekema E. J. (2016). Cryo-EM structure
of a tetrameric cyanobacterial photosystem I complex reveals novel
subunit interactions. Biochim. Biophys. Acta,
Bioenerg..

[83] Kargul J., Nield J., Barber J. (2003). Three-dimensional Reconstruction
of a Light-harvesting Complex I- Photosystem I (LHCI-PSI) Supercomplex
from the Green Alga *Chlamydomonas reinhardtii*: Insights
into Light Harvesting for PSI. J. Biol. Chem..

[84] Germano M., Yakushevska A. E., Keegstra W., van Gorkom H. J., Dekker J. P., Boekema E. J. (2002). Supramolecular organization of photosystem
I and light-harvesting complex I in *Chlamydomonas reinhardtii*. Febs Lett..

