# Supplementary material for: Structural Conservation of the A1 Binding Site in Photosystem I across Cyanobacteria and Green Algae
Source: ACS Omega. 2026 Mar 25;11(13):20605–15. doi: 10.1021/acsomega.5c12267 (PMC13068159; doi:10.1021/acsomega.5c12267)
Supplement: Supplementary file 1 [file ao5c12267_si_001.pdf]

Supplementary Information

To support

Structural Conservation of the A_1 Binding Site in Photosystem I Across Cyanobacteria and Green Algae

Gary Hastings[1,*], Hiroki Makita[2], Neva Agarwala[1], Michael R. Nelson[1], Julia S. Kirpich[1], Komalpreet Singh[1], Sreeja Parameswaran[1], Fedaa Ali[3], Barry D. Bruce[3,4], Haijun Liu[5], Lujun Luo[6], Wu Xu[6], Kevin Redding[7], Claudia Schade[8], Sarah M. Mäusle[8] and Dennis J. Nürnberg[8,9]

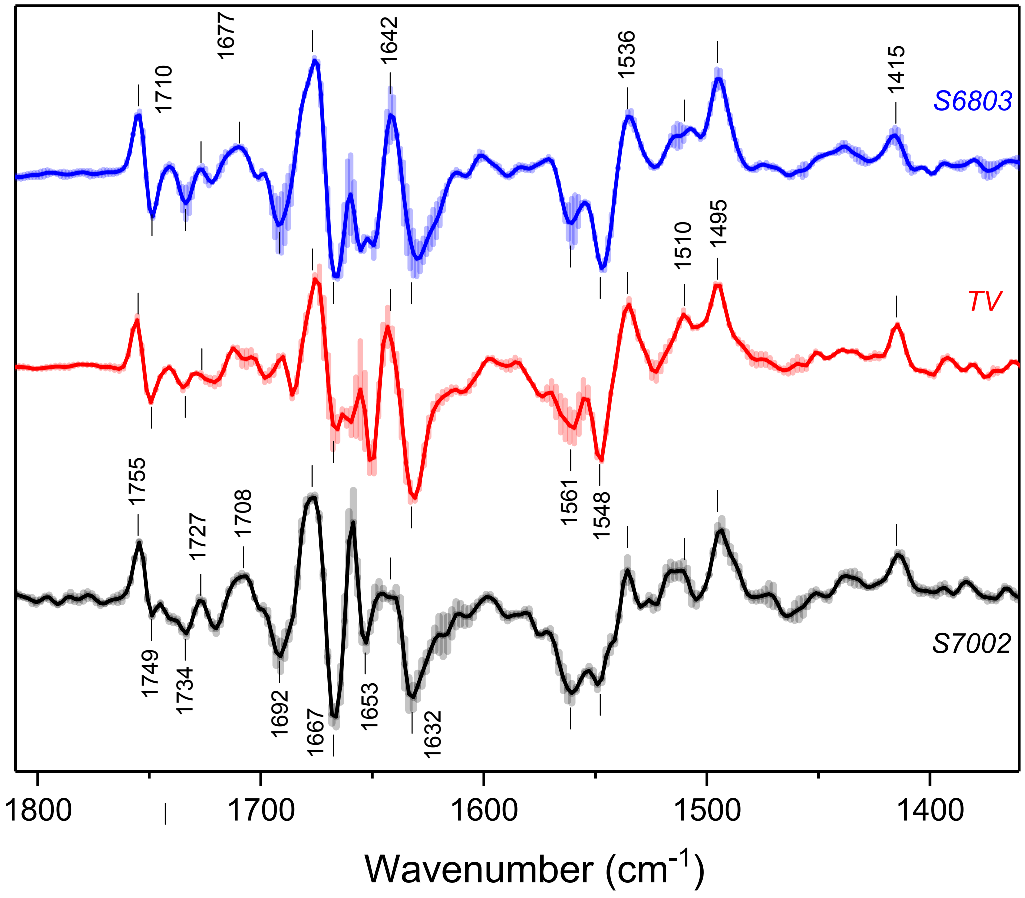

Fig. S1: Comparison of ($A_1^- - A_1$) FTIR DS obtained for PSI from *S6803* (*blue*), *TV* (*red*) and *S7002* (*black*). Error bars are shown for spectra that have been obtained in repeated experiments. For PSI from *S6803* and *TV*, DS for three samples from different labs were averaged. For *S7002*, samples are from three different FTIR DS measurements on PSI samples prepared at different times in our lab. The spectra are shown up to 1810 cm^{-1}, to demonstrate the noise level in regions where most molecular groups do not contribute.

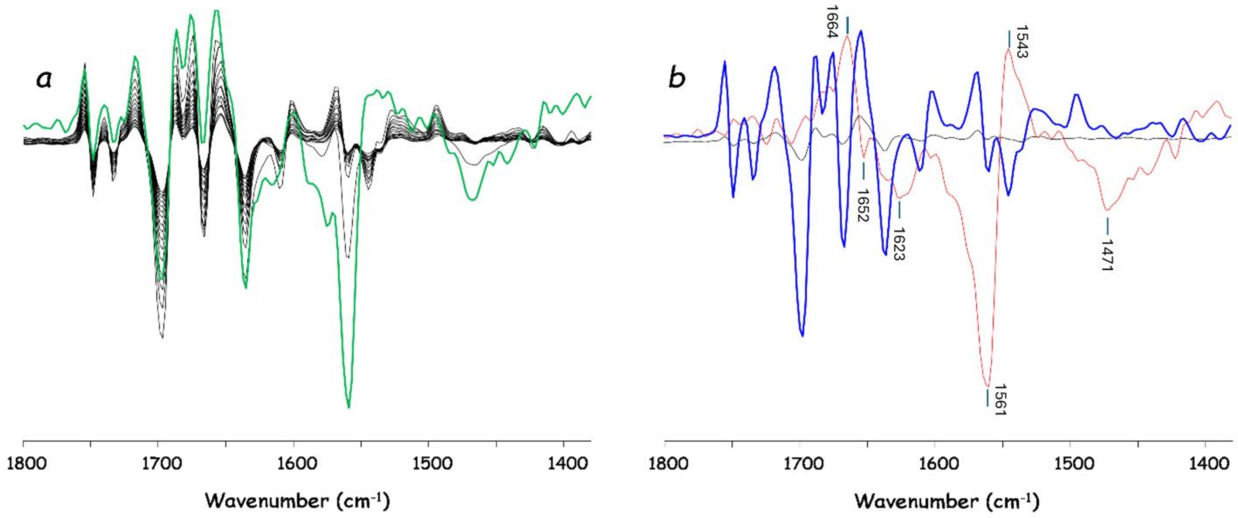

Fig. S2 (*a*) Time-resolved ($P700^+A_{1A}^-$ – $P700A_1$) FTIR DS in the 1800-1390 cm^{-1} region, obtained following laser flash excitation of *menB*⁻ PSI particles with PhQ incorporated into the A_1 binding site. Spectra at *t* = 0, 12, 54, 102, 150, 204, 252, 300, 248, 402, 450, 498, 552, 600, 648, 702, and 750 μs after the laser flash are shown. Except for the spectrum shown at t = 0 μs (*green*), the spectra shown are the average of three spectra centered at the indicated time point. So, for example, the spectrum at *t* = 12 μs is the average of spectra at *t* = 6, 12, and 18 μs.

From global analysis of all of the data obtained for PSI with PhQ incorporated, by fitting the data to a sum of two exponential phases and a non-decaying constant, three decay associated spectra (DAS) are obtained (*b*). A ~15 μs phase is obtained and is associated with a heating artifact related to laser flash excitation (*red*). A ~360 μs phase is obtained (*blue*) and is due to $P700^+A_1^-$ charge recombination. A DAS associated with the non-decaying component (with lifetime greatly exceeding the measurement timescale) is also shown (*black*).

Figure is reprinted in part with permission from Makita et al., *Biochimica et Biophysica Acta - Bioenergetics* **2017,** *1858* (9), 804-813 (Ref. 55 in manuscript). Copyright 2017, Elsevier Science &Technology Journals.

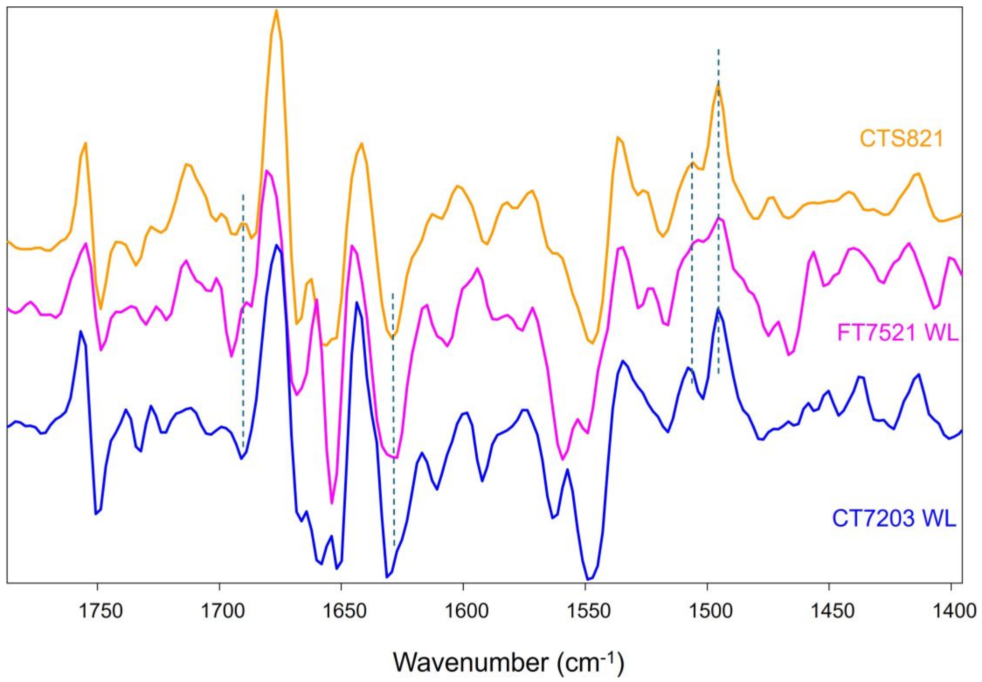

Fig. S3 Comparison of ($A_1^- - A_1$) FTIR DS obtained for PSI from *CTS821* (*orange*)*, FT7521* (*purple*) and *CT7203* (*blue*). Samples were from cells grown under WL.

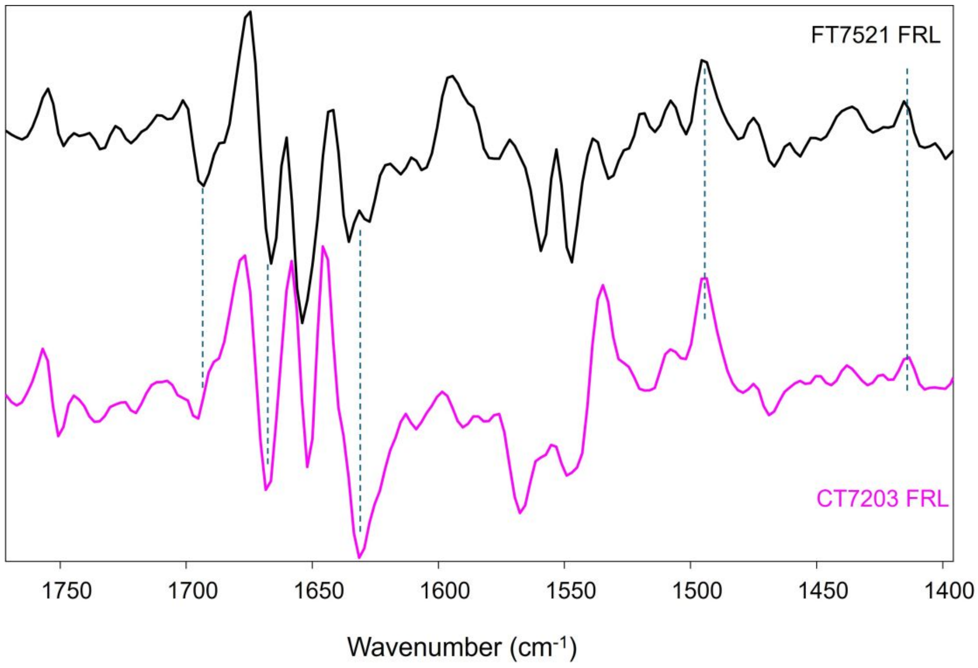

Fig. S4 Comparison of ($A_1^- - A_1$) FTIR DS obtained for FRL-PSI samples from *CT7203* (*purple*)*,* and *FT7521* (*black*).

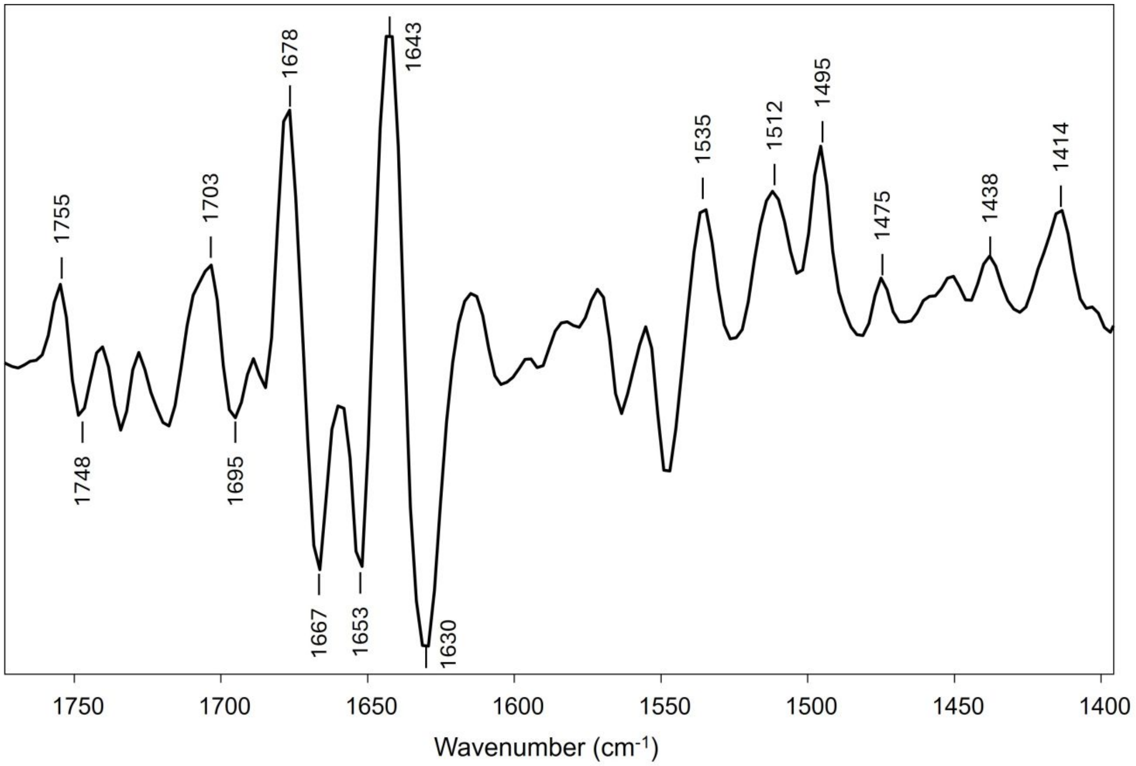

Fig. S5 ($A_1^- - A_1$) FTIR DS obtained for PSI from *CR*.
